# Inspection of Dairies

**Published:** 1895-08

**Authors:** J. H. Wattles


### INSPECTION OF DAIRIES.

BY J. H. WATTLES, V.S.

EDITOR JOURNAL OF COMPARATIVE MEDICINE.

DEAR SIR: In reply to your request for a short article for your July number, on the inspection of dairies that supply milk for use by the inhabitants of cities, I will say that to put the matter in the proper light it will be necessary to state a few of the conditions that exist, or have existed, in dairies where I have been called in my practice.

To the consumer of milk there has often come a peculiar odor, characteristic of new milk, that is usually considered as being one of the necessary elements of milk. My observation leads me to believe that nine-tenths of the so-called "animal odor" is caused by imperfect circulation in the skin of the animal, and by particles of dung falling into the pail of milk.

Cattle are milked in stables reeking with filth and with swarms of flies congregated upon the strainer that is placed upon the can, and the milk is filtered over the carcasses of drowned flies that may have just been feeding upon the sputa of a consumptive or regaling themselves upon other putrid or offensive matter, and their excrement is washed into the can of milk to be sold to the public.

Milk-cans are placed, after washing, in some exposed place near the stable to dry, and in one case the family outhouse was situated on one side of the drying-rack and one of the filthiest hen-houses one can imagine on the other side.

The sort of strainer used is usually one of tin with a wire-cloth mesh in the bottom of it, occasionally covered with either woollen or cotton cloth, but oftener without. The udder is frequently washed by dipping the hands into the milk that is in the pail or by milking into the hand and then washing the teat with the milk. Very seldom will one see the udder washed with clean water or wiped with a clean cloth. In one case, after the cow had put her foot into the pail half full of milk, the milk went in with the rest and the consumer was none the wiser.

A very small percentage of dairy farms are supplied with water of good quality and sufficient quantity. As a healthy cow will consume from sixty to one hundred pounds of water per diem, the influence that a supply deficient in quantity and quality would have upon the milk is obvious. It is not an uncommon thing to see the drainage from the stable passing into the stream from which the cows drink.

A cow confined to the stable requires a space of seven hundred and twenty square feet to obtain sufficient oxygen to aëreate the blood properly, but we usually see cattle huddled together in as close a space as possible, generally allowing each cow about two hundred and eighty square feet, where they are compelled to breathe and rebreathe the contaminated atmosphere because the dairyman informs us he can get more milk from them if they are kept close together.

The majority of stables are built as cheaply as possible, and are roughly finished inside and out, furnishing nooks and crannies for dirt to locate and disease to propagate. The floor frequently consists of two twelve-inch planks, laid side by side, just where the hind feet of the cows are placed and the milker stands when milking, with a trench about a foot wide behind the cows for the excrement to fall into. There is not one stable in a hundred that has any way of flushing, and no greater percentage has any system of drainage. There are stables that have stood for years upon the same ground and have contained hundreds of cows, and all the liquid manure made in them has been allowed to soak into the ground and there remain.

There has been so much said about milk containing germs of disease that it seems almost unnecessary to mention the fact, but we are so forcibly reminded that dirt and filth are the abiding places of disease and that cleanliness is the surest guide to health, that your attention is again called and you are again warned of the danger that exists.

No board of public health is complete without it has a thoroughly qualified veterinarian as a member. The physicians and other guardians of the public health are undoubtedly fully able to meet the conditions they are familiar with, but no man unless he has had a special training in the work is capable of filling the position ot inspector of dairies, inspector of milk, or inspector of meat.

Every city should pass an ordinance making it illegal to sell or offer for sale milk from a dairy that has not been inspected by a qualified veterinarian. The veterinarian should have absolute control of the sanitary surroundings of the dairy. He should be the one to say when and when not the water-supply, drainage, and ventilation are perfect, as well as to say when the cattle are healthy or unhealthy.

The veterinarian should be honest and fearless. He should feel that the dairyman is a citizen and entitled to all kindness and courtesy, and in making changes in the arrangement of stables and methods of handling the product should remember that he is in a position to be of great assistance or to work a cruel hardship; he should endeavor to make changes in a kindly manner, but with all the firmness that a public servant should have. No honest dairyman should or would object to a strict observance of sanitary measures, but the dishonest dairyman we must and will control.

In regard to the manner in which we can reach the dairy cows, I believe that every city should by ordinance order the milk-producer wishing to sell milk in the city to make application for inspection to the clerk of the board of health, and the clerk to issue an order to the veterinary inspector to examine such cows as are furnishing milk, and to make his return to the clerk of the board of health, the clerk to issue certificates according to the findings of the inspector, and to collect a fee for such certificate. In this manner the position would be made self-supporting, and the inspector would receive from the board of health such salary as would be determined upon, the fees collected going into a fund for such purposes.

In making our fight upon the present irrational system of milk inspection we must make every effort to convince our city officials that they are dealing with professional men, and we must remember that perhaps they are not aware of the strides forward that have been made by veterinarians in the past few years in the line of sanitation and inspection. We should by all means make the sanitary officers understand that we are not antagonizing them, but that we are antagonizing a system that is not placed upon a scientific basis.

The position should not be a political position, but should be filled by a competent man regardless of political belief or preference. The board of health should have control of dairy inspection, and all questions of difference between the inspector and dairyman should be adjusted by the board.

The general public are too well posted to be humbugged by any slipshod method of inspection, and the dealer in milk that has his supply shipped in, say, from ten to forty miles, is not doing himself justice unless he insists upon having every animal inspected from which he receives the milk. By inspection I do not mean that some broken-down politician be appointed or that some man be employed because he is cheap, but I mean that the inspector be paid enough to keep him from looking to the dairyman for perquisites, and that he be a man fitted to fill the position. As a business proposition, it seems to me that if a dealer was able to show to his customers certificates of examination of the cattle from which he derives his supply he could increase his business enough to pay him liberally.

A system of inspection that depends upon the bacteriologist to tell when cows are healthy or unhealthy, and to determine when milk is wholesome or unwholesome, is, to say the very least, ridiculous, and no bacteriologist of any standing whatever would have the impudence—it can be called nothing but impudence or ignorance—to claim that such methods of examination would be either practicable or reliable, as every authority one may select will testify.

The inspector should, if he is qualified to fill such an important position, be able to make all bacteriological tests, and a system that requires three men to perform the duties of one has the appearance of keeping two men to assist the third in doing nothing.

